# US Pediatric Inpatient Care Loss Before and During the COVID-19 Pandemic

**DOI:** 10.1001/jamanetworkopen.2024.46025

**Published:** 2024-11-22

**Authors:** Urbano L. França, Michael L. McManus

**Affiliations:** 1 Division of Critical Care, Department of Anesthesiology, Critical Care and Pain Medicine, Boston Children’s Hospital, Boston, Massachusetts; 2 Harvard Medical School, Boston, Massachusetts

## Abstract

This cross-sectional study compares the number of hospitals admitting children in 2021 compared with before the COVID-19 pandemic.

## Introduction

Early in the COVID-19 pandemic, many hospital pediatric units were repurposed to expand adult capacity in a restructuring process endorsed by the Children’s Hospital Association.^1^ During the pandemic, pediatric COVID-19 was mild, while school closures and social distancing reduced regular pediatric admissions, so remaining pediatric facilities were financially strained.^2^ The impact of this dynamic on access to pediatric hospital care has not been assessed, to our knowledge. In this study, we compare prepandemic with 2021 data to estimate changes in the number of hospitals admitting children.

## Methods

This retrospective, cross-sectional study was approved by the Boston Children’s Hospital Committee on Clinical Investigation and informed consent was waived because we used deidentified aggregate data. This study is reported in adherence to the Strengthening the Reporting of Observational Studies in Epidemiology (STROBE) reporting guideline.

We used 2019 and 2021 National Inpatient Sample (NIS) Databases from the Healthcare Cost and Utilization Project. The NIS draws from all Healthcare Cost and Utilization Project hospitals using a self-weighted, cross-sectional sample design that ensures representation across diverse factors, including hospital, census division, ownership, urban-rural location, teaching status, and bed size. We identified all hospitals with at least 1 pediatric (age <15 years) admission, excluding those caring only for infants younger than 1 year. Bootstrapping with 1000 iterations within each NIS strata set of admissions was used to estimate the medians and 95% CIs for the number of hospitals. Sensitivity analyses were performed by removing hospitals with 1 to 100 sampled admissions. Comparisons between hospital characteristics across years were assessed using the χ^2^ test. *P* values were 2-sided, and statistical significance was set at *P* ≤ .05. Analyses were conducted using Python version 3.10 (Python Software Foundation) from May to July 2024.

## Results

In the final dataset, 2169 hospitals had at least 1 pediatric admission in 2019 and 1795 hospitals had at least 1 pediatric admission in 2021, a reduction of 17.2% (*P* < .001). Almost half of this decline (181 of 374 hospitals [48.4%]) was in hospitals with more than 60% of admissions among Medicaid patients (Figure). These reductions were largest in number for hospitals that were private, nonprofit (206 of 374 [55.1%]); small (178 of 374 [47.6%]); or rural (182 of 374 [48.7%]). Compared with 2019, the largest proportional declines were in hospitals that were private, investor owned (297 to 217 [−26.9%]); small (748 to 570 [−23.8%]); or urban, nonteaching (411 to 276 [−32.8%]), with variability among the Census divisions (Table). Despite the overall decline, proportions within each of the characteristics remained stable, except for the increase in the proportion of urban teaching hospitals from 998 of 2169 hospitals (46.0%) to 941 of 1795 hospitals (52.4%) (*P* < .001).

**Figure.**
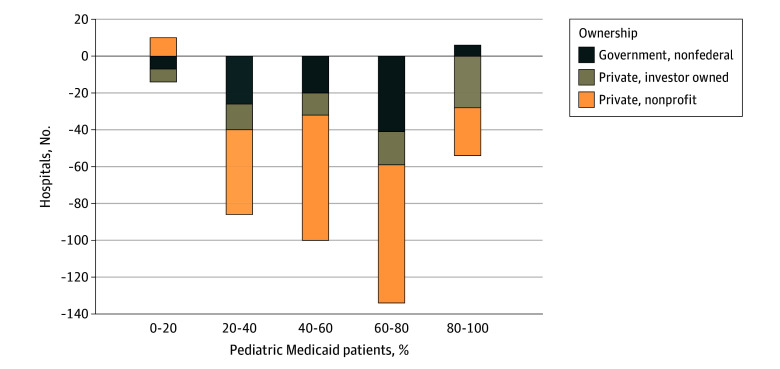
Difference in the Number of Hospitals Between 2019 and 2021 by Quintiles of the Fraction of Pediatric Medicaid Admissions and Hospital Ownership

**Table.** Hospitals Admitting Pediatric Patients by Characteristics in 2019 and 2021 and Percentage Change

Characteristic	Median (95% CI)^a^		2021 to 2019 difference, No. (%)	*P* value^b^
	2019	2021		
Total No. of hospitals	4564	4551	−13 (−0.3)	NA
Hospitals admitting pediatric patients	2169 (2150 to 2186)	1795 (1777 to 1814)	−374 (−17.2)	<.001
Pediatric Medicaid patients (quintiles), %				
0-20	256 (245 to 268)	252 (240 to 263)	−4 (−1.6)	.10
20-40	319 (315 to 321)	231 (228 to 233)	−88 (−27.6)	
40-60	606 (599 to 611)	506 (500 to 511)	−100 (−16.5)	
60-80	609 (605 to 612)	475 (471 to 477)	−134 (−22.0)	
80-100	379 (366 to 391)	332 (320 to 344)	−47 (−12.4)	
Ownership				
Government, nonfederal	392 (382 to 403)	303 (293 to 313)	−89 (−22.7)	.15
Private, investor-owned	297 (290 to 303)	217 (211 to 224)	−80 (−26.9)	
Private, nonprofit	1480 (1465 to 1493)	1274 (1260 to 1288)	−206 (−13.9)	
Size				
Small	748 (732 to 763)	570 (555 to 582)	−178 (−23.8)	.16
Medium	641 (632 to 648)	538 (528 to 548)	−103 (−16.1)	
Large	780 (773 to 787)	687 (679 to 694)	−93 (−11.9)	
Location				
Rural	760 (745 to 774)	578 (562 to 593)	−182 (−23.9)	<.001
Urban nonteaching	411 (402 to 420)	276 (268 to 284)	−135 (−32.8)	
Urban teaching	998 (990 to 1006)	941 (932 to 948)	−57 (−5.7)	
Division				
East North Central	319 (311 to 326)	248 (241 to 256)	−71 (−22.3)	.93
East South Central	141 (137 to 146)	119 (113 to 125)	−22 (−15.6)	
Middle Atlantic	216 (210 to 220)	189 (185 to 193)	−27 (−12.5)	
Mountain	203 (197 to 209)	155 (150 to 160)	−48 (−23.6)	
New England	86 (82 to 88)	72 (69 to 75)	−14 (−16.3)	
Pacific	268 (263 to 272)	246 (241 to 251)	−22 (−8.2)	
South Atlantic	337 (330 to 343)	271 (265 to 277)	−66 (−19.6)	
West North Central	275 (266 to 283)	221 (213 to 229)	−54 (−19.6)	
West South Central	324 (315 to 332)	271 (262 to 279)	−53 (−16.4)	

Abbreviation: NA, not applicable.

^a^ Medians and 95% CIs from 1000 iterations bootstrapping.

^b^ *P* values correspond to change in the proportion inside each characteristic.

## Discussion

Decades of consolidating pediatric hospital care have resulted in an increasingly concentrated system reliant on a small number of remaining facilities.^3^ Hospital consolidation, falling Medicaid reimbursement, and a decrease in volume of pediatric admissions have intensely pressured small and medium-sized hospitals, causing loss of pediatric services.^4,5,6^ Our observations suggest that this dynamic accelerated during the COVID-19 pandemic, with an additional loss of one-sixth of the nation’s remaining pediatric inpatient services. In number, this capacity decrease was most pronounced among small, rural, and private hospitals, with declines linearly proportional to Medicaid-insured pediatric admission fractions between 0% and 80%. Proportionally, the decline from 2019 was more pronounced in small, private or investor-owned, and urban nonteaching hospitals.

These findings are limited by NIS sampling methods, which are not stratified by patient characteristics and could miss some pediatric admissions. However, since sensitivity analyses yielded similar results, the observed reductions likely represent conservative estimates within the CIs provided. Second, since the NIS does not include short-stay or observation admissions, some small hospitals serving these could be missed. Third, since COVID-19 spikes continued in 2021, some hospitals may have still been actively prioritizing adults.

Rapid, uncontrolled closure of pediatric hospital services presents a public health challenge. While some closed pediatric inpatient services may reopen, history and system dynamics suggest that most will become permanent casualties of the pandemic. If so, access to care among vulnerable rural and Medicaid populations will be especially affected.

## Notes

Supplement.
Data Sharing Statement
