# Supplementary material for: US Pediatric Inpatient Care Loss Before and During the COVID-19 Pandemic
Source: JAMA Netw Open. 2024 Nov 22;7(11):e2446025. doi: 10.1001/jamanetworkopen.2024.46025 (PMC11584919; doi:10.1001/jamanetworkopen.2024.46025)
Supplement: Supplement. — Data Sharing Statement [file jamanetwopen-e2446025-s001.pdf]

Data Sharing Statement

França. US Pediatric Inpatient Care Loss Before and During the COVID-19 Pandemic. *JAMA Netw Open*. Published November 22, 2024. doi:10.1001/jamanetworkopen.2024.46025

Data
Data available: No

Additional Information
Explanation for why data not available: HCUP NIS data is available for purchase at https://hcup-us.ahrq.gov/tech_assist/centdist.jsp
